# Atrial Fibrillation: The Science behind Its Defiance

**DOI:** 10.14336/AD.2016.0211

**Published:** 2016-10-01

**Authors:** Maureen E. Czick, Christine L. Shapter, David I. Silverman

**Affiliations:** ^1^Department of Anesthesiology,; ^2^Department of Psychiatry, Hartford Hospital/Institute of Living, and; ^3^Echocardiography Laboratory, Hartford Hospital, Hartford, CT 06106, USA.

**Keywords:** atrial fibrillation, pathophysiology, catheter ablation, surgical maze procedure, antiarrhythmic drugs, autonomic imbalance

## Abstract

Atrial fibrillation (AF) is the most prevalent arrhythmia in the world, due both to its tenacious treatment resistance, and to the tremendous number of risk factors that set the stage for the atria to fibrillate. Cardiopulmonary, behavioral, and psychological risk factors generate electrical and structural alterations of the atria that promote reentry and wavebreak. These culminate in fibrillation once atrial ectopic beats set the arrhythmia process in motion. There is growing evidence that chronic stress can physically alter the emotion centers of the limbic system, changing their input to the hypothalamic-limbic-autonomic network that regulates autonomic outflow. This leads to imbalance of the parasympathetic and sympathetic nervous systems, most often in favor of sympathetic overactivation. Autonomic imbalance acts as a driving force behind the atrial ectopy and reentry that promote AF. Careful study of AF pathophysiology can illuminate the means that enable AF to elude both pharmacological control and surgical cure, by revealing ways in which antiarrhythmic drugs and surgical and ablation procedures may paradoxically promote fibrillation. Understanding AF pathophysiology can also help clarify the mechanisms by which emerging modalities aiming to correct autonomic imbalance, such as renal sympathetic denervation, may offer potential to better control this arrhythmia. Finally, growing evidence supports lifestyle modification approaches as adjuncts to improve AF control.

For many medical professional’s atrial fibrillation may bring to mind a slight twist on a famous quote by late US Supreme Court Justice Potter Stewart: “I can’t explain atrial fibrillation, but I know it when I see it.” Although the pathophysiology of atrial fibrillation (AF) may seem somewhat impenetrable, the arrhythmia itself is diagnostically all but unmistakable, so most medical professionals would indeed “know it when they see it.” And they see it with alarming frequency as AF is such a pervasive problem; it is the most common cardiac arrhythmia across the globe [1 - 3], causing 1/3 of all arrhythmia-related hospitalizations [4]. The 2010 Global Burden of Disease study reported 33.5 million AF cases worldwide [5]. The ATRIA study estimated that 2.3 million people in the US alone had AF in 2001 [6]; by 2009, that number was above 3 million [7]. With the aging of the post-war “baby-boom” generation [3, 8], over 5 million Americans are projected to have AF by the year 2050 [9], although some projections take that number much higher [3].

The high prevalence of AF is partly due to the treatment resistance of this arrhythmia -- often it may seem subdued by medical interventions, only to resurge again. Refractoriness to therapy, combined with the tremendous number of risk factors that drive AF occurrence, and the increased longevity of patients with cardiovascular disease in general, have yielded a steadily expanding pool of AF cases.

Odds of developing AF increase dramatically with aging: from age 40 onward, the lifetime risk is 26% [3]; above age 60, prevalence doubles with the passage of each additional decade of life [10]. Men are more likely to develop AF than women [11, 12]. Incidence varies between racial groups, with whites more frequently affected than blacks [13]. And some families may carry inherited genetic predisposition for AF [14 - 16].

Higher rates of AF occur in extreme endurance athletes [17], and with many medical problems including hypertension [18, 19], gastroesophageal reflux (GERD) [20], asthma [21, 22], chronic obstructive pulmonary disease (COPD) [23, 24], hemodialysis [25 - 27], diabetes [28], anxiety and depression [29], mitral regurgitation [30], and stenosis [31, 32]. 10-15% of patients with hyperthyroidism will develop AF [33]. And 1/3 of congestive heart failure patients manifest AF [1], together with 30% of those who undergo bypass surgery for coronary artery disease [34 - 36], up to 20% of those who undergo lobectomy for lung cancer, and 40% of those who have full pneumonectomy [37].

Obesity increases the risk of AF 49% [38], with a 4% rise in incidence for each 1-point BMI elevation above normal [8]. Sleep apnea, despite its association with obesity and hypertension, is an independent risk factor for AF [39, 40]. Roughly ½ of AF patients have sleep apnea [39, 40], and AF is up to 18 times more likely to initiate within 90 seconds of an apneic hypoxic episode [41].

Alcoholic beverages [42, 43], cigarettes [44], nonsteroidal anti-inflammatory drugs (NSAIDs) [45], and theophylline [46], a stimulant found in black and green tea, sometimes used as a pulmonary medication, are reported to increase AF incidence. Interestingly though, caffeine, which was long assumed to be proarrhythmic, appears to have been exonerated from triggering AF in both the Framingham Study [47] and the Women’s Health Study [48]. In fact, low-dose caffeine may even be protective against AF [49, 50].

In almost ¾ of cases, AF occurs with risk factors as mentioned above [51, 52], but it can also sometimes rear its head in younger patients, without any known risk factors [53]. Genetic predisposition may play an important role in such cases of “lone AF” [14 - 16].

Even more alarming than the pervasiveness of the risk factors are the potentially catastrophic complications. Coexisting AF doubles mortality in patients with coronary artery disease [54], doubles risk of dementia [55], triples risk for heart failure [55], and quintuples risk for stroke [3, 54].

Given the impact of AF, it is not surprising that enormous resources are poured into the effort to suppress or cure this arrhythmia. Unfortunately though, AF remains defiant, stubbornly refractory to pharmacological and surgical attempts at management. Much has been discovered about the underlying pathophysiology of AF and this can shed light on why so many risk factors predispose to it. But more importantly, understanding AF’s underlying cellular mechanisms can reveal why AF has so successfully eluded cure, and can point toward future approaches that may prove more successful at curbing this tenacious arrhythmia.

## Deconstructing the Pathophysiology of AF

Decades of research have yielded alternative hypotheses for the mechanism underlying AF [56]. The focal source hypothesis [57] argues that a single area of rapid ectopic action potential firing can bombard the atria with so many electrical impulses that the resulting chaos initiates fibrillation [58]. The multiple wavelet hypothesis posits that several simultaneous self-sustaining waves of abnormal conduction, called reentry loops, emit an array of depolarizing prompts in all directions, leading to fibrillation [59].

Newer experimental data suggest that features of both theories might synergize [60], yielding a hybrid mechanism of AF, in which a focal source of ectopy initiates a solitary reentry wave which electrically shatters, in a process called wavebreak, to trigger fibrillation.

### STEP 1: ECTOPY

All atrial cells do not repolarize at the exact same moment [58, 61]. Autonomic innervation of the heart is punctate [1, 62], so some atrial cells will inevitably be located closer to autonomic nerves and will experience a higher concentration of autonomic neurotransmitters than neighboring cells. This results in small differences in degrees of neurotransmitter-mediated channel modification, yielding slight differences in channel conductance and therefore slight repolarization time disparities. In the normal heart, repolarization heterogeneities “all come out in the wash.” Every cell repolarizes before the SA node re-depolarizes them during the following cardiac cycle.

But, if an atrial cell misbehaves, launching its own ectopic action potential, then repolarization disparities become enormously significant [53]. The electrical impulse propagating from the ectopic beat would find some atrial cells with slower repolarization speeds refractory to conducting another action potential at that time. Refractory cells provide areas of unidirectional conduction block [63], deflecting away the depolarization impulse created by the ectopic beat. But nearby atrial cells with slightly faster repolarization speeds would be able to use the ectopic electrical impulse to launch their own action potentials. In this manner, an ectopic beat can set in motion an aberrant depolarization wave, triggering action potentials in any cells in its path that are recovered and ready to fire again [53].

### STEP 2: REENTRY

If conduction velocity of the aberrant depolarization wave is slow enough, the wave’s path length is long enough, and refractory periods of originally blocked cells are brief enough, then the aberrant wave may loop around and discover that the previously blocked cells have finished repolarizing, and can now fire and incorporate into the wave. If the wave survives this long, it may circle back on itself a second time, re-encountering its earliest participant cells, and triggering action potentials in those cells yet again. In this way, the depolarization wave may establish a reentry conduction loop that can sustain itself potentially indefinitely [52, 64].

Alternatively, if the conduction of the wave is too quick or the refractory periods of the originally blocked cells are too long, then the wave will run out of cells to depolarize, and will dissipate rather than entrenching in a sustained loop [65, 66].

### STEP 3: WAVEBREAK

When an ectopic beat does trigger reentry, AF may onset when the reentry wave encounters an area of abrupt conduction slowing -- either functional slowing, due to slight variations in individual cells’ ion channel conductance, or structural slowing due to fibrosis. Like an ocean wave slamming into a rock jetty, the electrical wave shatters into uncountable wavelets that fan out in all directions to trigger the disorganized atrial electrical hyperactivation of AF [52, 56, 67].

This mechanism explains the onset of AF, but to understand what makes onset possible in the first place, it is necessary to examine how AF risk factors create the substrate that triggers ectopy and enables reentry.

## Promoting Ectopy

**Figure 1.**
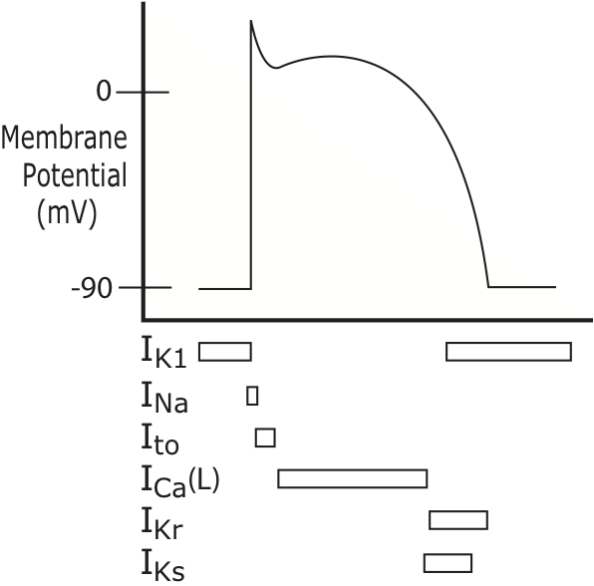
**Atrial Action Potential Ion Currents**. While the SA cells are depolarizing, the surrounding contractile cells of the atria are at their resting membrane potential of approximately -90mV, due to the IK1 current. Once the SA depolarizes, it promptly passes depolarizing positive ions to the atrial cells through low resistance gap junction channels. These positive ions bring the atrial cells to their own threshold potential, opening voltage-gated sodium channels (INa) in the atrial cell membranes, so that the atrial cells fire their own action potentials. At the peak of the upstroke in the atria, transient outward (Ito) potassium channels open; positively-charged potassium ions exit the cell, beginning the process of repolarization. Their attempt to repolarize the atrial cells is short-lived however, because inward calcium current, conducted through voltage-gated L-type calcium channels (Ica(L)) keeps the cells in a state of depolarization just a bit longer, depicted as a plateau in the middle of the action potential waveform. The SA action potential does not need a calcium-based plateau current because SA cells are not responsible for contracting. Atrial cells, on the other hand, use the electrical depolarization from the action potential as the signal to contract. The “trigger” calcium entry through L-type channels during the plateau acts as a bridge between the electrical depolarization and mechanical contraction. The L-type channels inactivate rapidly, calcium current ceases, and then potassium exit, through multiple channels including the “ultra-rapid”-opening IKur, the “rapid” opening IKr (also called hERG channels) and the “slowly” opening IKs channels, fully repolarizes the cells.

**Figure 2.**
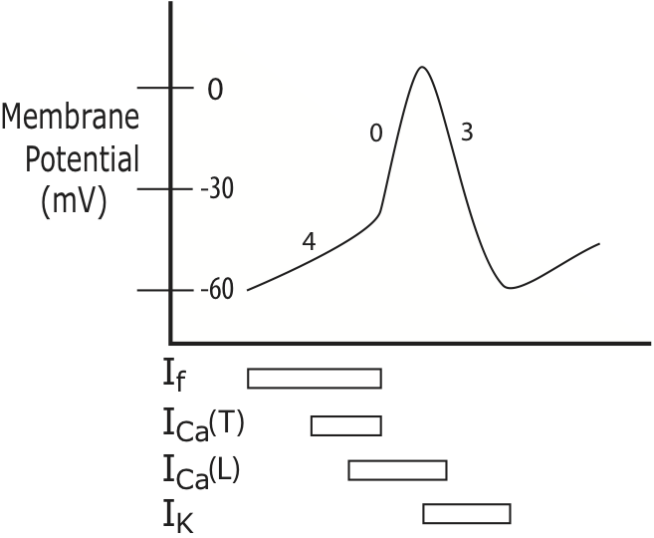
**Sinoatrial Node Depolarization**. Under normal conditions, the heart’s electrical rhythm is generated by the cells of the sinoatrial (SA) node. At the beginning of each cardiac cycle the membrane potential of the SA cells is approximately -60 mV, with the interior of the SA cells negatively charged relative to the cell exterior. Unlike contractile cardiac cells, SA cells do not have a stable resting membrane potential, so they remain poised at -60 for just the briefest moment, because “funny channels” (If) promptly spring open, allowing positively charged ions to leak from the extracellular space into the interior of the SA cells. As positive ions enter, the SA cell interiors become progressively less negatively charged (depolarized). The funny channel leak current (soon joined by Ca2+ current through T- and L-type channels, ICa(T), ICa(L)) -- and the change in the membrane potential that results from it -- is represented in the graph of the SA node action potential as the diagonal upslope at the start of action potential waveform, also referred to as “phase 4.” The positive ion influx quickly brings the SA cells toward the “threshold potential,” at approximately -40 mV, at which point voltage-gated calcium channels suddenly open, enabling a sudden massive surge of positive charge entry into the cell. This is the upstroke of the SA action potential, also called “phase 0.” Following the upstroke, there is an exodus of positively-charged potassium ions (IK) which restores the cell interior to its original negatively-charged baseline electrical potential during phase 3 repolarization.

In contracting atrial cells the very substantial IK1 current, which largely determines the resting membrane potential, swamps out the effects of the funny current leak channels, so that atrial cells do not normally display automaticity [2]. (Fig. 1) The SA node, unencumbered by IK1, initiates each cardiac cycle by self-depolarizing and then passing the depolarization wave to the remainder of the atrial cells [63, 68, 69]. (Fig. 2)

However, if the SA node becomes dysfunctional or the funny current depolarization rate of a latent pacemaker accelerates [65] in response to increased sympathetic or decreased vagal drive to the heart [65, 70], an alternative site in the atria may “outrun” the SA node [53] and fire an ectopic beat, via abnormal automaticity.

Ectopic beats can also occur by triggered activity: abnormal depolarization events occurring in between the firing of two consecutive action potentials [65]. One triggered activity variant, called early afterdepolarizations, EADs, can occur if action potential repolarization is prolonged [52], because of genetic mutation of one of the repolarizing K^+^ channels (in Long QT Syndrome) [71], K^+^ channel blocking drugs, acidosis or hypokalemia, all of which diminish the repolarizing K+ current. Slow repolarization can allow the L-type Ca^2+^ channels to reopen a second time, creating an inward positive current spike, the EAD, during repolarization.

The other triggered activity variant, delayed afterdepolarizations, DADs, occur with cardiac cell Ca^2+^ overload. During diastole, ryanodine receptors should stay closed and Ca^2+^ ions should remain within the sarcoplasmic reticulum (SR) and not enter the myocardial cell cytosol. However, if the SR becomes overfilled, some Ca^2+^ can escape into the cytosol [52] during diastole, triggering inappropriate activation of the contractile apparatus, and impairing diastolic relaxation. To preserve chamber relaxation and filling, “diastolic Ca^2+^” is promptly removed from the cell by the Na^+^/Ca^2+^ exchanger [52], which brings 3 Na^+^ ions into the cell for each solitary Ca^2+^ ion pumped out. This 3+ in: 2+ out stoichiometry yields a net +1 inward current that creates the DAD, a positive spike in the membrane potential after action potential repolarization has already concluded [2]. If sufficient in size, a DAD or EAD can take the cell back to threshold and launch an ectopic action potential [52].

Where the pulmonary veins (PVs) meet the left atrium (LA), “sleeves” of atrial muscle cells extend to envelop the blood vessels [56, 72], possibly acting as an external valve system, preventing backflow of blood into the PVs during atrial contraction [73]. The sleeve cells receive extensive innervation from parasympathetic [72] and sympathetic [74] nerves, and they have histological similarities to the pacemaker cells of the SA node [73]. Likely due to these factors, the PV sleeves can emit focal ectopy, possibly by abnormal automaticity given the cells’ similarity to SA cells, but also potentially due to EADs [75], making this vascular-cardiac interface a frequent initiation source for lone AF [76 - 78]. A similar situation has been noted in the right side of the heart, around the vena cava-right atrial junction [79]. Thyroid hormone has been reported to augment ectopic activity by PV cells, which begins to explain AF promotion by hyperthyroidism [80].

However, over 70% of AF cases are not lone, but instead are associated with cardiopulmonary disease [51, 52]. In this larger subgroup, focal ectopy may come from DADs due to abnormal Ca^2+^ handling. Angiotensin II, upregulated in hypertension, promotes ryanodine receptor phosphorylation, potentially contributing to SR Ca^2+^ overload [52]. Coronary artery disease is known to increase Na^+^/Ca^2+^ exchanger function, also a player in generation of DADs [52]. In heart failure, the SR can become Ca^2+^ overloaded by digitalis glycosides [65], which inhibit the Na^+^/K^+^ ATPase pump, triggering the Na^+^/Ca^2+^ exchanger to run “in reverse,” moving Na^+^ ions out and bringing additional Ca^2+^ into the cell to improve cardiac contractility, yet creating risk for DADs [52].

SR Ca^2+^ overload can also occur during very rapid heart rates, including persistent sinus tachycardia or reentry tachycardias [81], which allow larger amounts of Ca^2+^ to enter, due to increased frequency of L-type channel openings [81]. (Fig. 1) In addition, with increased sympathetic tone (typical during tachycardias and a frequent feature of cardiac disease), elevated norepinephrine levels mediate higher degrees of L-type channel phosphorylation, increasing channel conductance and Ca^2+^ entry, which then can promote DADs [82].

## Promoting Reentry

### Slow conduction and long path length

Voltage-gated sodium channels mediating the action potential upstroke require the cell to fully repolarize in order to reset their gates. In partially repolarized cells, some sodium channels fail to reset and thereby remain off-line which functionally slows conduction. If these partially repolarized cells are re-excited by an ectopic beat or aberrant depolarization wave, their slower conduction increases the likelihood that reentry will be established [65]. Hyperkalemia [65] and the NSAID ibuprofen [83] have also been shown to promote reentry by functionally slowing conduction.

Anatomic disturbances, such as scar tissue from a healed myocardial infarction site or fibrosis due to aging, will disrupt myocardial cell contact points, slowing conduction [65]. Conduction is fastest down the long axis of a cardiac myofibril [65]. When a wavefront must round a curve, to accommodate small veins or changes in myocardial fibril orientation, then conduction slows. The PV/LA junction has irregular fibril orientations that can delay conduction and support reentry [81, 84, 85], further enhancing its ability to act as a source of lone AF.

Atrial dilation -- from cytotoxicity due to excess alcohol use [86], or from volume-pressure overload of the atria caused by diastolic dysfunction [87], extreme endurance training [88], valve regurgitation [30] or stenosis [31] -- provides longer pathlength for a propagating wavefront, promoting reentry. Dilation also increases atrial chamber diameter, augmenting wall stress. This triggers compensatory collagen deposition in the extracellular matrix to strengthen the wall [87], but it also slows conduction which facilitates reentry.

### Short refractory period and conduction block

Atrial stretch from volume-pressure overload shortens action potential duration, secondary to altered K^+^ channel expression [89]. In addition, PV/LA junction cells exhibit greater shortening of action potential refractory periods in response to vagal stimulation than do the other left atrial cells [56, 90, 91], creating neighboring areas of unequal repolarization that promote conduction block and reentry.

Hyperthyroidism promotes AF by shortening action potential duration [33, 92]. Hyperthyroidism has been reported to exert more pronounced action potential shortening in cells of the right atrium, relative to those of the left, augmenting conduction heterogeneities that favor conduction block and reentry [92, 93].

Most genetic mutations implicated in familial AF increase the activity of K^+^ currents and thereby shorten action potential duration and refractory period [14]. An inherited reentry predisposition such as this correlates with clinically-observed data from the Framingham Study, which revealed significantly increased risk of AF in study participants whose parent(s) also had AF [15, 94].

Inflammation has been implicated in promoting AF [95, 96], particularly in the setting of cardiac surgery [36, 97, 98]. Although post-bypass AF is likely multifactorial, local inflammation from surgical cannulation of the atrium has been reported to exacerbate atrial conduction heterogeneities [98, 99], which may facilitate reentry.

Both branches of the autonomic nervous system have reentry-promoting potential. The sympathetic transmitter norepinephrine shortens refractory period by increasing the IKs current [100], (Fig. 1) because faster heart rates mandate a shorter repolarization time, in order to fit more action potentials into a given time frame. Acetylcholine (ACh) from parasympathetic nerves opens ACh-sensitive K^+^ channels, generating IKACh current to speed up action potential repolarization [65]. This is likely a protective mechanism: if vagally-mediated slower heart rates actually prolonged repolarization time, then EADs could be promoted, leading to arrhythmia. There is mounting evidence that imbalance between the two arms of the autonomic nervous system plays an important role in AF [1, 75, 81, 101].

## Autonomic Imbalance

Autonomic imbalance has been reported in many conditions linked to AF, including obesity [102], sleep apnea [103, 104], depression [105], diabetes [106], asthma [107], cardiovascular disease [108], heart failure [109, 110], and extreme endurance activities [111 - 113]. Animal models provide supporting evidence: in dogs, intravenous epinephrine or acetylcholine enabled AF induction in 21% and 100% of the animals respectively [101, 114]. Again in dogs, augmented sympathetic tone, via electrical stimulation of either stellate ganglion, was shown to increase incidence of AF; surgical removal of either ganglion decreased rates of fibrillation [115].

AF is postulated to have two autonomic subtypes: vagally-predominant, in younger patients, with onset typically at night when vagal tone is higher, and adrenergically-predominant, in older patients, with episodes more common during daytime when sympathetic tone is at its circadian peak [116, 117]. Episodes of paroxysmal lone AF, originating from the pulmonary veins, have been reported to begin after a shift toward higher vagal tone [118]. But in persistent AF, increased sympathetic tone was detected, correlating with fewer parasympathetic neurons but increased numbers of sympathetic neurons in cardiac nerve bundles innervating the atria [56, 119, 120]. One study reported that significant abrupt sympathetic stimulation, in the presence of previously elevated vagal tone, could elicit focal ectopy [121]. Other studies report the opposite pattern, elevated sympathetic tone followed by abrupt increase in parasympathetic tone, preceding AF onset [101, 122]. Thus the interplay between the two autonomic divisions might be even more important than their individual roles [75], rendering appropriate balance between them critical. But since 70% of AF cases correlate with risk factors [51, 52], sympathetic predominance is the more widespread problem.

Sympathetic overactivation is a well-established risk factor for cardiac morbidity and mortality [123, 124], since the cardiovascular system is essentially trapped in the high-energy-utilizing sympathetic-dominant mode, leading to premature “burnout” of the system [125]. Sympathetic overactivation may be driven by many factors, including obesity and cardiovascular dysfunction [126], but there is also evidence to suggest that chronic stress may play a role [127].

Autonomic preganglionic neurons receive regulatory input from a web of higher brain centers called the central autonomic network [128], to appropriately deploy autonomic drive during basal conditions, or to modulate autonomic activity during a stress response to threat or injury [129, 130]. The network includes the paraventricular nucleus (PVN) of the hypothalamus, an integration hub for homeostasis and stress response functions [131]; the rostroventrolateral medulla (RVLM), whose pre-sympathetic pacemaker neurons provide tonic sympathetic drive [132]; and the nucleus tractus solitarius (NTS) in the medulla, which integrates peripheral reflexes, including the baroreflex and chemoreflex, to modulate autonomic outflow [128, 133].

In addition to cathecholamine- and cortisol-modulated cardiorespiratory responses to immediate threat, survival requires learning to avoid future danger [134 - 137], so emotion/memory centers of the limbic system, including the amygdala, are integrated into the central autonomic network, to activate fight-or-flight responses [138, 139]. The stress response is intended to be short-term [129]; chronic cortisol elevation from chronic stress alters the amygdala, producing dendrite hypertrophy and hyperexcitability [140 - 142], which may alter limbic contribution to the network [143] to then alter sympathetic drive [144].

Although it appears that there are no published articles directly linking AF with hypercortisolism, indirect evidence does exist. There is a clear link between hypercortisolism and physical or psychological stressors [145, 146]. Furthermore, human and animal studies reveal autonomic imbalance associated with multiple psychological stressors that can provoke anxiety and depression [147 - 150]. Anxiety and depression in turn have been reported to increase AF incidence [29] and AF recurrence rates after cardioversion [151] and ablation [152] procedures.

## Exploring Treament Resistance

The broad range of risk factors detailed above plays an important role in making AF the most common arrhythmia in the world [153], but there is another side to the story. Once AF starts, it quickly becomes harder and harder to stop, because its pathophysiology becomes self-reinforcing, altering the atria to make them much more likely to continue fibrillating. The pathophysiology of AF can now be used as a lens to bring into clearer focus the means that have enabled AF to evade pharmacological suppression and surgical or interventional attempts at cure. And it can provide insight into emerging approaches that may more effectively silence this tenacious arrhythmia.

## Entrenching AF: Electrical and Structural Remodeling

In the 1990s, Wijffels and colleagues coined the phrase “atrial fibrillation begets atrial fibrillation” [154], indicating that AF causes electrical, structural, and autonomic alterations of the atria which make the atria more likely to remain in AF. This atrial remodeling appears to play a role in the clinical progression of AF from paroxysmal to persistent to permanent [155] and in AF’s resistance to treatment [96].

Autonomic remodeling briefly appeared in the discussion of autonomic imbalance: in persistent AF, fewer parasympathetic and increased sympathetic nerves innervate the atria [120]. However, this is a later step in the remodeling process; the earliest atrial alteration due to AF is electrical remodeling. During rapid heart rates characteristic of AF, atrial myocytes attempt to shield themselves from the toxic effects of excess Ca^2+^ entry by reducing L-type Ca^2+^ current amplitude [81]. Unfortunately this narrows the action potential plateau [70], and shortens refractory period, thereby promoting reentry. Thus the atrial cells’ attempt to protect themselves from Ca^2+^ overload paradoxically perpetuates AF. In tachypacing animal models, evidence of electrical remodeling appears on day one of the arrhythmia [64]. In goats subjected to burst pacing, the atrial effective refractory period decreased by 35% within the first 24 hours and took almost a week to resolve after the arrhythmia ceased, creating a prolonged window of time with increased susceptibility to recurrence [64, 156, 157].

AF-induced physical alteration of the atria, called structural remodeling, follows suit [81]. Rapid heart rates [64] and chronic volume overload from ineffective atrial emptying during AF [52] cause atrial dilation and fibrosis [64]. These atrial changes further promote reentry by increasing pathlength and slowing conduction. Although contractile cardiac myocytes take up 75% of the cell volume of the heart, they are outnumbered by fibroblasts [158, 159]. During development, fibroblasts build the cardiac skeleton, which maintains the heart’s structural integrity [158, 159]. With normal aging, but exacerbated by pathological conditions including heart failure [160] and hypertension [161], cardiac fibroblast content and collagen crosslinking increase [158], initiating a progressive decline in cardiac relaxation [87] that perturbs pressure-volume relationships. The age-related atrial geometry alteration and impaired conduction promote reentry and provide substrate for wavebreak, helping illuminate the powerful correlation between AF and aging: in Framingham study participants, AF prevalence was 2% between age 60 and 69, but rose to 5% between age 70 and 79, and 9% between age 80 and 89 [162, 163].

Angiotensin II activates NADPH oxidase which promotes fibrosis by generating reactive oxygen species [155], playing a role in the increased incidence of AF in hypertension. Chronic sympathetic overstimulation also increases fibroblast proliferation and promotes cardiac structural remodeling [158, 159], contributing to AF in a wide array of conditions including heart failure [110] and sleep apnea [132, 164]. As a result, autonomic imbalance, which helps initiate AF, also helps to maintain AF over the long term, via structural remodeling.

Some studies suggest that inflammation may play a causative role in the initiation of AF. For instance, mice that overexpress Tumor Necrosis Factor (TNF) alpha have increased likelihood of AF [96]. And in a dog pericarditis model, suppression of inflammation by corticosteroids significantly decreased AF incidence [96]. Increased levels of the inflammatory mediator C-Reactive Protein (CRP) correlate with increased likelihood of developing AF [95, 96, 165]. However, it is important to note that CRP levels have been shown to decline after cessation of AF upon successful radiofrequency ablation procedure [95, 166]. This latter finding suggests that increased inflammation might be the result of AF, rather than the cause.

While a role for inflammation in the original onset of AF remains subject to debate, inflammation does appear to play a significant part in AF persistence [96]; for example, continually elevated CRP levels correlate with increased likelihood of AF recurrence after cardioversion [95, 167]. Promotion of structural remodeling by inflammatory mediators has been suggested as the mechanism for this: higher levels of pro-fibrotic TNF alpha [95, 168] and CRP [96, 169] have been found in persistent AF, compared to paroxysmal AF.

In the subset of post-cardiac surgery AF patients, evidence is the most convincing that acute inflammation, from surgical trauma to the atria and exposure of blood to extracorporeal circulation, does play a role in triggering AF onset [36, 95, 98]. Elevated CRP levels after cardiac surgery were predictive of increased post-operative AF incidence [96, 170]. Moreover prophylactic corticosteroids help to reduce, although not eliminate, the arrhythmia in this population [171]. However, even in post-surgery patients, AF remains most likely to occur in patients with prior risk factors that alter atrial structural and electrical properties and thereby promote fibrillation, including age-related fibrotic changes, diastolic dysfunction, and atrial dilation [36, 98].

## Antiarrhythmic Drugs

While remodeling contributes markedly to AF treatment resistance, some aspects of the treatment regimens themselves also limit their effectiveness. The Vaughn Williams system classifies antiarrhythmic drugs according to their primary target channel. But many of these drugs have crossover effects at multiple channels [172, 173], yielding combined arrhythmia-suppressing and arrhythmia-promoting effects (as well as potentially dangerous side effects) [174] which contribute to the low overall success rate of these drugs against AF [162].

Class Ia drugs procainamide [175] and quinidine [176], and class Ic drug flecainide [177], suppress reentry by a secondary channel effect, inhibiting IKr (the hERG channel), which results in elongation of refractory period. However, the primary effect of these drugs, inhibition of INa, actually promotes reentry by slowing conduction velocity [174]. Although prolonging repolarization in the atria confers some protection from AF, the very same effect in the ventricles prolongs the QT interval, putting patients at risk for torsades, which can lead to ventricular fibrillation [178]. The proarrhythmic effects of class I drugs increase mortality, by triggering lethal ventricular arrhythmias, particularly in post-infarction patients [179, 180] and in those with reduced left ventricular function [181]. A meta-analysis assessing quinidine for sinus rhythm maintenance after cardioversion of AF found that use of the drug increased death rates [182]. Similarly, flecainide was shown to increase ventricular tachycardia and ventricular fibrillation when used for AF suppression [178]. As a result, class Ic drugs are now restricted by AHA guidelines to AF patients with at least near-normal left ventricular systolic function, age below 75 years and no known ischemic coronary disease [183].

Beta-blockers, Vaughn Williams class II antiarrhythmics, are used to blunt the rapid ventricular response to AF, due to their ability to slow AV Node conduction. On paper, these drugs should have some arrhythmia suppression potential, by blocking norepinephrine-mediated augmentation of IKs and ICa currents [81], and the resultant action potential narrowing. However, this does not translate into clinically significant suppression of AF, except after cardiac surgery, when beta-blockers have been shown to significantly reduce post-operative AF [36, 184 - 186]. Class IV agents, L-type Ca2+ channel blockers, are also used to blunt the ventricular rate response to AF, but are reported to prolong AF episodes [174, 187], probably because L-channel blockade shortens refractory period. These drugs have not been shown effective for AF suppression [188, 189].

Class III agents inhibit K+ currents, particularly IKr, so they delay repolarization and prolong the refractory period, which resists reentry [53]. Sotalol came to market as a racemic mixture combining D-isomer anti-reentry class III effects with L-isomer class II beta-blocking effects [174], but its class III QT prolongation effect was found to increase mortality in post-infarction patients [190]. A retrospective review found sotalol superior to conventional beta-blockers in preventing post-cardiac surgery AF [191], but it was demonstrated inferior to amiodarone in maintaining sinus rhythm after electrical cardioversion [174].

Early studies suggested that dofetilide, regarded as a pure class III agent [192], was superior to placeibo [193, 194] and to sotalol [194] for achieving pharmacological cardioversion of AF to sinus rhythm, and for maintaining sinus rhythm after cardioversion [192 - 194]. A later study confirmed dofetilide’s efficacy for cardioversion but also reported that initial cardioversion success was not predictive of long-term sinus rhythm maintenance [195]. Successful conversion to sinus rhythm is less likely in longer-duration AF cases [192], consistent with the role of atrial remodeling in AF treatment refractoriness. Dofetilide therapy must begin in-hospital, with continuous cardiac monitoring, due to its QT prolongation effect [195]; in the DIAMOND-CHF study, greater than 3% of dofetilide recipients developed torsades [196]. Rates of torsades appear to be lower when dofetilide dosing is adjusted based upon creatinine clearance, as the drug is approximately 80% renally excreted [197].

Amiodarone has effects within all four Vaughn Williams classes, so it has arrhythmia-promoting and suppressing potential. It inhibits INa, beta receptors, IKr, IKs, Ito, IK1, and ICa-L [174], but the effects of the drug apparently evolve over time, with class I and IV effects present early and class III effects becoming more prominent through long-term use [174, 198]. The class III effects make amiodarone the most successful drug available to suppress AF [174], decreasing AF after cardiac surgery by meta-analysis [199], and decreasing arrhythmia-related death in randomized trials [198]. However, amiodarone’s efficacy is severely limited by its side-effect profile, which forces 20-50% of patients to discontinue its use [198]. Amiodarone can precipitate digoxin toxic reactions [198], and it inhibits metabolism of warfarin derivatives, exacerbating their anticoagulant effect [198]. Amiodarone’s high iodine content [198] can interfere with multiple steps in the synthesis and release of thyroid hormone, leading to either hypo- or hyper-thyroidism [200]. Up to 30% of amiodarone patients develop neurological side effects including tremor, ataxia, neuropathy and dizziness [198]. And up to 25% will develop abnormalities in liver function tests [198]. Perhaps most catastrophically, amiodarone can trigger chemical pneumonitis that can evolve into pulmonary fibrosis [198], carrying a mortality rate as high as 33% with late recognition, but still 10% with early recognition [198].

Given the risks of antiarrhythmic drugs, multiple randomized trials have attempted to determine whether or not these drugs conferred benefit over rate-control strategies [81]. Trials included RACE, AFFIRM, STAF, and HOT-CAFÉ in patients with normal left ventricular function, as well as DIAMOND-CHF and AF-CHF in those with heart failure. No trial has revealed survival advantage with any of the anti-arrhythmic drugs evaluated [201 - 203], and subgroup analysis of AFFIRM may suggest decreased survival in certain patient subsets, such as the elderly and those with coronary disease [204]. Furthermore rate-control and rhythm-control groups had equivalent rates of stroke occurrence, stressing the necessity of continued prophylactic anticoagulation while utilizing anti-arrhythmic medications in AF patients [81].

## The Surgical Maze

Because of the risks and side-effects of rhythm-control drugs, as well as their poor overall efficacy against AF, surgical alternatives were explored, hoping to cure AF by altering atrial anatomic characteristics [205, 206]. Of these, the maze procedure, developed by James Cox in the late 1980s, gained the most widespread application. Maze surgery consists of cutting then re-sewing a series of location-specific, full-thickness incisions through the walls of both atria. The resulting scars create a maze-like pattern of “blind alleys,” capable of conducting sinus rhythm, but theorized to block AF propagation.

With the original version of the surgery, now called maze I, “free of AF” rates in greater than 90% of patients were reported in case series [207 - 209]. But significant complications prompted serial adaptations to the maze procedure, yielding maze II, and then maze III [210, 211]. Published success rates for maze III range widely: from 64% free of AF at 4 years [212], to 96% [213], or even 98% [214], at 5+ years of follow-up.

Commentators have argued that success rates for the maze may be overestimated [206] because studies lacked rigorous means to document post-procedure AF recurrences [215]. Some maze case series report AF recurrence rates based on follow-up phone calls, questionnaires, or single EKGs alone [206, 215]. Random EKG checks could miss episodes of paroxysmal AF, and since 1/3 of patients do not recognize when they are in AF [216], phone questionnaires would not be reliable indicators. Other maze studies do not report AF return rates at all, but define success via post-procedure stroke rates [206]. Accurate evidence of post-maze AF recurrences would require longer-term monitoring, but even this may suffer from inaccuracies due to potential patient non-compliance [206].

Maroto and colleagues showed that early post-operative recurrence of AF after open-heart maze procedure, in this case utilizing radiofrequency ablation rather than cut-and-sew incisions, is a risk factor for late recurrence [217]. In this study, 59% of patients experienced early post-operative AF recurrence; and within three years, 32% had AF return [217]. Specific patient characteristics increase the likelihood of AF return after the maze [205, 218, 219], including increased left atrial diameter, particularly if greater than 6cm, and longer duration of pre-procedure AF with greater remodeling and fibrosis. Thus, factors that promote reentry and wavebreak seem to make the surgical maze less likely to succeed in the long-term. In addition, scar lines from the maze could be expected to slow conduction and provide substrate for reentry and wavebreak. This is significant because the maze often does not stop a patient from having episodes of the atrial reentry arrhythmia that originally instigated AF [220]; there is a body of literature on radiofrequency ablations for atrial tachycardia and atrial flutter after maze surgery [218, 221]. The persistence of post-maze atrial reentry arrhythmias potentially opens the door to wavebreak-driven AF recurrences in the future. Progression of underlying cardiac arrhythmia substrate, including fibrosis due to aging, autonomic imbalance, or ongoing cardiac disease might render a post-maze patient, who early on seemed to be cured, once again susceptible to AF. Given this, patients are often advised to continue prophylactic anticoagulation.

The classical surgical maze is performed via median sternotomy and cardiopulmonary bypass which carry risks, especially in already-compromised patients. Modifications to make the maze less invasive have been developed, including thoracoscopic approach, but results suggest similar limitations to the original [205]. The maze technique carries its own risks [205, 222], including a persisting decrease in atrial contractility [223], and 10-19% reported requirement for permanent pacemaker because of SA node dysfunction, after even maze III [214, 222, 224] since classical maze incisions disrupt SA arterial supply [225]. Because of these drawbacks, surgical maze is rarely offered to patients with lone AF, and is largely performed on patients who are undergoing open-heart surgery for some other reason, such as coronary artery occlusive disease or valve dysfunction [205].

## Radiofrequency Catheter Ablation

For nonsurgical AF candidates, intravascular catheter ablation is increasingly utilized, and meta-analyses have shown it to be more successful against AF than anti-arrhythmic drugs [226, 227]. Unlike the uniform lesion set performed on all maze recipients [205], catheter ablation employs electrophysiological mapping to customize each procedure to the patient’s individual arrhythmia substrate. Mapping has yielded ablation success rates above 90% for a variety of atrial arrhythmias including atrial tachycardia, AV Node reentry tachycardia, AV reentry tachycardia and atrial flutter [228].

The mainstay of ablation therapy for AF is electrical isolation of the pulmonary veins behind scar lines. But for AF, in contrast to other atrial tachyarrhythmias, published catheter ablation success rates are typically quoted as 50-70% [228, 229]. A 2005 international survey of ablation centers [230] reported a 52% AF ablation success rate, but with 27% of patients requiring more than one AF ablation procedure [229]. A 2012 review of the California State Inpatient Database evaluated 4,156 patients who received a first AF ablation procedure between 2005 and 2008, reporting readmission rates for AF recurrence of 21.7% by 1 year and 29.6% by 2 years [231]. Radiofrequency ablators deliver high frequency current to destroy cardiac cells in contact with the catheter tip, creating lesions with a central nidus of necrosis, surrounded by areas of inflammation [228]. The donut of inflamed tissue around the necrotic center may not be able to conduct arrhythmia immediately after the ablation, but later it may recover conduction, enabling AF recurrence [228].

The complexity of the reentry-wavebreak pathophysiology may also drive post-ablation AF recurrence. Radiofrequency ablation of AF often does not terminate the original reentry tachycardia that triggered fibrillation [232]. And 30-50% of patients develop new atrial tachycardias after extensive ablation for AF [233 - 236]. These reentry arrhythmias, together with conduction slowing from ablation scar lines have been implicated [236] in AF return. Given these data, post-ablation patients are often advised to continue anticoagulation.

A 2014 editorial pointed out that the majority of AF ablation cases represented in published trials were younger patients with paroxysmal rather than persistent AF, and without structural heart disease [229]. Such patients have the least severe AF substrate and constitute a comparatively small percentage of the larger spectrum of AF cases. Evidence suggests that, just as with the maze, ablation is less successful in more complex AF [237] with extensive remodeling, for example in sleep apnea patients [238, 239], and those with structural heart disease. The ongoing CABANA trial may in the near future shed clearer light on ablation success rates across the full spectrum of more complex AF patients [229].

At present the use of catheter ablation for AF remains the subject of intense debate [229, 240], and is best reserved for highly symptomatic patients who have failed multiple attempts at cardioversion and/or drug suppression. The procedure itself carries risk, including valvular insufficiency [241], left atrial thrombus [242], atrial-esophageal fistula [243], and pulmonary vein stenosis if radiofrequency energy is applied too close to the ostia of the veins [228, 244]. In addition there is 0.5% risk of damage to the conduction system requiring permanent pacemaker placement, 1-2% risk of cardiac perforation/tamponade, 2-4% risk of vascular damage due to percutaneous vascular access [228], and a 4.7% reported incidence of periprocedural stroke [231].

## New Directions

Procedures targeting autonomic imbalance have begun to be utilized in animal models and preliminary human studies of AF. In dogs, low-level vagal stimulation was reported to decrease AF inducibility in response to focal ectopy generated by high-frequency, sympathetic-mimicking atrial stimulation during the atrial refractory period [245]. Also in animals, radiofrequency ablation of the cardiac autonomic ganglion plexus, to halt unequal outflow of autonomic stimulation to the heart, has been reported to increase the success of ablation procedures in eliminating AF [246]. However, in human patients, studies of combined ganglion plexus destruction and pulmonary vein isolation by radiofrequency ablation have yielded mixed results in preventing AF recurrence [75, 247, 248]. Moreover, such extreme disruption of cardiac autonomic input might have deleterious hemodynamic consequences in compromised hearts, for in animal models of heart failure, ganglion blockade can cause death [249].

In dogs, renal sympathetic denervation, which diminishes central sympathetic drive rather than abolishing cardiac autonomic input, decreased AF incidence after electrical stimulation of the stellate ganglion [250]. In a sleep apnea model, renal denervation decreased frequency and duration of AF, by blunting apnea-induced shortening of atrial refractory period [251]. In a small study of 27 patients, pulmonary vein isolation plus renal denervation reported improved freedom from AF at 1 year compared to pulmonary vein isolation alone [252]. Larger renal denervation trials are underway [253].

A few studies have begun to assess effectiveness of integrative medicine and lifestyle approaches in suppressing AF. The LEGACY trial recently revealed that sustained weight loss decreased AF burden, by Holter monitor and symptom scoring, over 5 years of follow-up [254]. Obesity is associated with a persistent low-grade pro-inflammatory state [255-258], as well as with sympathetic overactivation [259, 260]. Weight loss has been shown to improve autonomic balance and decrease inflammation [261-263], which would be expected to lower their contributions to AF promotion.

Acupuncture, which has been shown to enhance parasympathetic tone [264, 265], has been reported to reduce AF burden, however results thus far have come from small numbers of patients. A small 2012 study showed that acupuncture significantly reduced number and duration of AF episodes [266]. A 2011 study reported that acupuncture decreased recurrences after radiofrequency ablation of persistent AF [267]. And a 2013 case report documented improved retention of sinus rhythm after AF cardioversion in a patient with chronic pulmonary disease [268]. Promotion of improved autonomic balance may underpin these effects on AF burden.

In the Yoga My Heart study, yoga was reported to decrease paroxysmal AF episodes and decrease resting heart rate in a single-center 3-month-long study, utilizing symptom diaries and event recorders [269]. Both yoga and acupuncture have been reported effective in treating post-traumatic stress disorder (PTSD), so it appears that both modalities have the ability to modulate the limbic system [270 - 272], and potentially alter its contribution to autonomic imbalance. In addition, both yoga and acupuncture have been reported to lower markers of inflammation, providing another potential avenue for the reported decrease in AF burden [273 - 275].

## Conclusion

Atrial fibrillation continues to grow in prevalence worldwide [7], despite pharmacological, surgical and interventional efforts to suppress it. The underlying pathophysiology of AF illuminates the reasons behind its treatment refractoriness. AF begins remodeling the atria on the first day of onset, making the atria much more likely to continue fibrillating. Many anti-arrhythmic drugs have arrhythmia-promoting potential, which diminishes their effectiveness. Scarring from maze and ablation procedures may sometimes facilitate reentry and wavebreak, enabling AF recurrence. Although results are preliminary, lifestyle modification and autonomic rebalancing approaches may provide the key to more successful control of AF in the future.
